# Sexuality behind clinic doors: experiences of healthcare professionals in primary care addressing sexuality with older people

**DOI:** 10.1093/ageing/afag219

**Published:** 2026-07-27

**Authors:** Andrea Kocurkova, Danielle De Boos, Heather Cogger-Ward

**Affiliations:** School of Psychology, Sport Science & Wellbeing, College of Health and Science, University of Lincoln, Brayford Pool, Lincoln LN6 7TS, United Kingdom of Great Britain and Northern Ireland; University of Nottingham Faculty of Medicine and Health Sciences, Doctorate in Clinical Psychology, School of Medicine, University of Nottingham, B Floor, YANG Fujia Building, Jubilee Campus, Wollaton Road, Nottingham NG8 1BB, United Kingdom of Great Britain and Northern Ireland; Doctorate in Clinical Psychology, School of Medicine, University of Nottingham, B Floor, YANG Fujia Building, Jubilee Campus, Wollaton Road, Nottingham NG8 1BB, United Kingdom of Great Britain and Northern Ireland

**Keywords:** sexuality, older adults, primary care, qualitative research, healthcare professionals, older people

## Abstract

**Background:**

Sexual health in later life remains an overlooked area in healthcare. Despite being the first point of contact for most patients, primary care healthcare professionals (HCPs) rarely initiate discussions about sexuality with older adults (OAs). This study investigates how these HCPs talk about sexuality in later life and examines the extent to which existing guidelines support inclusive, age-appropriate care.

**Objective:**

To explore NHS primary care professionals’ experiences of discussing sexuality with OAs, including the circumstances that prompt these conversations, the factors shaping them and the extent to which professionals use formal guidance in these discussions.

**Design:**

A qualitative design was used. Semi-structured interviews were conducted with 16 HCPs in England. An inductive reflexive thematic analysis approach was used for data analysis.

**Results:**

Three key themes were identified. First, unspoken assumptions, clinician discomfort and time pressures often limited discussions, creating uncertainty around how and when to address sexual health. Second, physical symptoms served as ‘natural openers,’ but such conversations maintained their medical focus, overlooking other aspects contributing to OAs’ quality of life. Third, participants described minimal reliance on guidance, instead drawing on instinct or experience. Overall, discussions were reactive and shaped by competing clinical demands. Clinicians consistently expressed a need for clear, concise and psychologically informed tools and training.

**Conclusion:**

This study highlights the need to normalise later-life sexual health discussions by attending to clinician discomfort and integrating inclusive practices into routine care. Recognising sexuality as important in later life may help challenge age-related assumptions in primary care.

## Key Points

- Primary care discussions about sexuality with older adults are usually reactive rather than routine.
- Clinician discomfort, time pressure and assumptions about ageing often limit these conversations.
- Physical symptoms act as common triggers for discussing sexual health, but broader intimacy and wellbeing are often overlooked.
- Primary care professionals report limited use of formal guidance and rely largely on personal experience or instinct.

## Introduction

Western societies are experiencing demographic change, with declining birth rates and increased life expectancy contributing to an ageing population [1]. While healthcare advancements have extended life expectancy, clinical attention remains focused on disease management, often overlooking broader aspects of wellbeing, including sexuality [2]. The World Health Organisation (WHO) defines sexuality as central to human life that extends beyond physical acts to include gender identity, emotional intimacy, interpersonal relationships and social roles [3]. Nevertheless, sexuality in older adults (OAs) remains neglected in medical and wider social discourse [4–6], which may be reinforced by stereotypes of OAs as asexual, despite evidence that many remain sexually active in later life [7–10].

Both healthcare professionals (HCPs) and OAs often hesitate to discuss sexual health [2, 11–14]. Many OAs expect clinicians to initiate these conversations, creating a ‘do not ask, do not tell’ dynamic [2, 6, 15]. This mutual silence may contribute to under-diagnosed dysfunction and decreased quality of life, affecting intimacy, relationships and emotional wellbeing [6, 13, 15], reflecting a persistent gap in support for OA’s sexual health identified in a recent systematic review [16].

HCPs commonly report uncertainty or discomfort in addressing sexuality with OAs, citing limited expertise, training, time pressures and concerns about causing offence [12, 17, 18]. Differences in age, gender or sexual orientation between clinician and patient may further shape these difficulties [19]. These barriers suggest that conversations about sexuality depend not only on clinicians’ knowledge, but also on their confidence to raise sensitive topics. This aligns with the concept of self-efficacy, defined here as clinicians’ belief in their ability to discuss sexuality with OAs in practice [20]. Low self-efficacy may contribute to avoidance when clinicians feel underprepared, unsupported or constrained by time-limited consultations [20, 21].

Without sufficient training, experiential learning and peer support, clinicians’ self-efficacy [20] may be undermined, limiting the extent to which guidelines translate into clinical practice. Consistent with this, a systematic review of sexual-health education interventions found wide variation in medical and nursing curricula, suggesting that many future clinicians lack the skills to address OA sexuality [22]. Under time pressure [21], clinicians may rely on heuristics or intuitive judgements [23] that reinforce assumptions about later-life sexuality, despite contrary evidence [7, 10].

These assumptions are not neutral and may carry gendered implications. Older women may face particular disadvantage, as their concerns are more likely to be dismissed, medicalised or treated as a natural part of ageing [18, 24, 25]. Such patterns reflect gendered ageism and medical misogyny [24], and may place the onus on older women to self-advocate in complex healthcare environments [26, 27]. Health systems that define women’s sexuality through reproduction tend to deprioritise their sexual wellbeing post-menopause [28], while pharmacological interventions for older men’s sexual function are more readily offered [8]. Socioeconomic disadvantage can further exacerbate these inequities, with some OAs experiencing increased stigma and reduced service access, reflecting wider patterns of healthcare exclusion and health inequality [29].

Although patients often prefer to receive sexual-health advice from their usual primary care provider [13], clinician beliefs, training and comfort ultimately shape the care OAs receive [12, 13, 19]. Clinical guidance can support and normalise age-inclusive sexual-health discussions, but existing recommendations are limited, inconsistently applied and age-neutral [11, 14, 30]. While the National Institute for Health and Care Excellence (NICE) offers broad sexual health guidance, it does not offer actionable, age-specific support [31].

Frameworks, such as Permission, Limited Information, Specific Suggestions and Intensive Therapy (PLISSIT) [32], offer practical tools for sexual health consultation; however, evidence of their routine use remains limited [12]. Rising sexually transmitted infection (STI) rates amongst OAs [33] further highlight the need for age-inclusive, evidence-based guidance. Normalising conversations about sexuality in later life, therefore, requires addressing systemic barriers and strengthening clinicians’ confidence through training, experiential learning and supportive environments [33].

As the most frequently accessed healthcare setting, primary care is uniquely positioned to support sexuality in OAs. The 2025 GP Patient Survey reported that 77% of adults aged ≥55 had consulted their GP practice within the previous 6 months [34], emphasising its role in person-centred and preventative care, aligning with the National Health Service (NHS) Long Term Plan [35]. Integrating sexual health into routine primary care may improve wellbeing and quality of life [2, 12].

Further research is needed to understand how primary care HCPs navigate sexuality conversations with OAs in practice, including the barriers and facilitators that shape these discussions. This study examines how HCPs in primary care approach sexuality with OAs, the role of clinical guidelines and the informal strategies used when guidance is absent or inconsistently applied.

## Aims

1. To explore how HCPs in primary care navigate discussions about sexuality with OAs, examining the factors that influence these conversations and the role of professional guidelines in shaping clinical practice.

### Secondary aims

1. To examine the circumstances when discussions about sexuality with OAs occur and the reasons why they may be omitted.
2. To assess the role of professional guidelines in supporting HCPs in addressing sexual health with OAs by examining awareness, relevance and applicability of existing guidelines in primary care consultations.

## Method

### Design

This study employed a qualitative approach, using semi-structured interviews. Ethical approval was granted by the Health Research Authority (23/HRA/5000). The study was informed by a critical realist epistemology, which recognises the existence of an objective reality while acknowledging that knowledge of it is shaped by social and contextual factors [36].

### Recruitment and participants

HCPs working in NHS England primary care, aged ≥18, who regularly saw patients aged ≥55 and might discuss sexuality with them, were invited to take part in a one-to-one interview. Recruitment used convenience, purposive and snowball sampling through online dissemination, including social media groups on Facebook, Reddit and X targeting UK-based primary care communities (e.g. r/GPUK and r/NursingUK), to include HCPs across roles and experience levels [37, 38], as shown in Table 1. Participants were encouraged to share advertisements within their professional networks. Prospective participants completed an online screening questionnaire to assess eligibility, gather demographic and professional background information and obtain informed consent.

**Table 1:** Research participant demographics.

	(*N* = 16) *N* (%)
**Sex**	
Male	5 (31%)
Female	11 (69%)
**Age**	
Mean (*SD*)	36.88 (10.01)
Minimum - maximum	27–62
**Experience (years)**	
Mean (*SD*)	9.81 (7.65)
Minimum - maximum	2–30
**Profession**	
GP	6 (37.5%)
Mental health practitioner	4 (25%)
GP registrar	3 (18.75%)
Mental health nurse	2 (12.5%)
Nurse assistant	1 (6.25%)

Online recruitment on Reddit, where an inconvenience allowance was mentioned, generated several dozen suspected fraudulent responses. Suspicious emails were identified based on characteristics described in recent literature, including clustered timing, vague wording, similar email formats and copied study advert text [39], and were excluded to maintain research integrity.

### Data collection

Sixteen semi-structured interviews were conducted remotely via Microsoft Teams by the first author, who had no prior relationship with the participants. Informed consent was obtained beforehand and participants were reminded of confidentiality limits, their right not to answer questions and secure data handling. Transcripts were anonymised and pseudonyms were used in reporting. The interview schedule used broad, open-ended questions covering participants’ understanding of later-life sexuality, when it arose in consultations, perceived barriers and facilitators and guideline awareness [40], key interview topics were shared in advance alongside the informed consent. Interviews lasted 27–60 minutes. Sexuality was conceptualised per the WHO definition [3], though terminology in reporting reflects participants’ own language, which typically focused on clinical presentations such as erectile dysfunction or menopause.

### Data analysis

The first author anonymised and transcribed all audio-recorded interviews verbatim. Data were analysed inductively using reflexive thematic analysis (RTA), following Braun and Clarke’s six-phase approach [41]. Recognising RTA’s recursive nature, transcripts were reviewed for developing themes as interviews progressed, with analytical sufficiency rather than data saturation as the stopping criterion [42]. Sufficiency appeared by interview 12 and 4 further interviews were completed to broaden perspectives and to confirm the stability of themes [42, 43].

Coding was inductive and semantic, with codes generated iteratively and refined across transcripts. The first author coded all interviews, with initial codes reviewed, refined and, where appropriate, merged into broader patterns, which were developed into themes with latent meanings explored. Subthemes were used only where they added analytical clarity. Developing interpretations were discussed regularly with supervisors to refine theme boundaries and ensure findings remained grounded in the data.

### Quality assurance

Several strategies were used based on standard practices for qualitative rigour [43]. These included reflexive journaling, regular supervision and an audit trail of analytical decisions. Credibility was supported through systematic cross-checking of themes against the original transcripts and by using Braun and Clarke’s 15-point checklist for thematic analysis [44]. This framework supported the iterative coding and re-coding process through to the final report, ensuring that themes were grounded in the data, internally coherent and analytically distinct [44].

Throughout, critical reflection and documentation were used to maintain researcher reflexivity and enhance analytical rigour [45]. Reflexive notes and supervision supported consideration of how the first author’s clinical psychology training may have shaped attention to discomfort, silence and relational dynamics.

## Results

Sixteen HCPs participated in the study. Participant characteristics are summarised in Table 1.

### Results of the RTA

Results from the RTA indicated three key themes shown in Table 2. Subthemes were used where they helped organise distinct aspects of a theme. Theme 2 is presented without subthemes as it captured one clear pattern in data.

**Table 2:** Table of themes.

Themes	Subthemes
Navigating sensitive conversations: the interplay of comfort and discomfort	What’s left unspoken: the silent walls around sexual health
	Building bridges to disclosure
Natural openers: how physical symptoms invite sexual health conversations	-
Guidelines in the shadows: navigating the gaps between formal guidance and real-world practice	Learning in the trenches: how experience shapes conversations
	The gaps we feel and guidelines on autopilot

### Theme description

#### Theme 1: navigating sensitive conversations: the interplay of comfort and discomfort

This theme, explored across two subthemes, captures the unspoken barriers to discussing sexual health, while also highlighting strategies that helped support disclosure and trust.

**What’s left unspoken: the silent walls around sexual health**. Conversations about sexuality in later life often remained unspoken, shaped by HCPs’ assumptions about OAs’ sexuality. Many participants described assumptions that OAs were asexual or less interested in sexuality, particularly older women after menopause or bereavement. These beliefs acted as a ‘silent wall’, limiting when and how HCPs asked about sexuality:

We don’t like to ask them questions because we have a perception of the older generation that they don’t have a sexuality or they don’t engage in sexual contacts. (*Daisy, 33, Senior MH Practitioner*^1^)

Unlike physical symptoms, such as pain or medication side-effects, which were more routinely explored in clinical interactions, most participants described sexuality concerns as absent unless explicitly raised by either the patient or clinician. Without this initiation, sexuality was often omitted from medical discourse, reinforcing the idea that it was no longer relevant in later life. Systemic barriers, such as time constraints and competing clinical priorities, further contributed to this silence:

I do find it hard because of the time restraint that we have; we do have to take a history, examine and make a management plan all within 10 minutes. So, it can be hard to think about exploring sexual health questions. Especially in the context of […] the elderly population […]. There are other things that are important for us to rule out. *(Ivy, 33, GP)*

Several participants explained that OA consultations focused on chronic illness management and unless sexual health was linked to an urgent medical issue, it was sidelined. Some younger female participants expressed caution when sexual health was raised by older male patients, especially if uncertain about patients’ motivations and how such conversations might be interpreted:

[…] do have especially in the mental health field you do get the over 55 male patients of a perverse nature then, yeah, I would be uncomfortable. *(Amelia, 38, Senior MH Practitioner)*

Rather than fostering open dialogue, some accounts suggested that intimacy and emotional needs could become framed as issues to be managed or redirected, particularly where clinicians were uncertain how to respond within the boundaries of routine care:

Help her to change her perspective on it and to just see it from a different way […] she’s obviously 80 now and she’s still got those emotions and feelings […] how do you […] stop her emotions at that age of 80 in case she starts falling in love with someone else? *(Heidi, 31, GP)*

This perspective suggests that expressions of intimacy in later life could be interpreted as issues to manage rather than recognised as a legitimate aspect of ageing. Participants suggested that systemic constraints and functional models of care contributed to this invisibility, more through omission than intent, leaving many OAs unsupported in navigating intimacy.

**Building bridges to disclosure.** Several participants emphasised creating space for patients to voice and normalise concerns and described normalising sexual health as one way of reducing embarrassment:

But then so in amongst other things, I’d probably say to people: ‘Sorry to ask this I ask everybody,’ you’re normalising it then, aren’t you? *(Charlie, 35, Senior MH Practitioner)*

This approach helped shift conversations from taboo to routine, making sensitive questions feel less unexpected or intrusive. Participants also described reassurance as an active strategy for signalling that sensitive disclosures were welcome and legitimate:

I’ll just be open with them and say: ‘You know, I am asking you them questions because I want to let you know that this is a safe space and that you are able to discuss anything that you haven’t been able to do in the past […]’ (Daisy, 33, Senior MH Practitioner)

These strategies were identified by several participants as informal yet effective ways to foster trust and manage emotional vulnerability. Over time, several participants reported that such relational approaches could dismantle the ‘silent walls’ that surrounded sexual health in OAs.

#### Theme 2: natural openers: how physical symptoms invite sexual health conversations

All participants described physical symptoms as the main gateway through which sexual health surfaced in consultations. For many OAs, these concerns were not presented as standalone issues but emerged alongside broader medical problems and were therefore more likely to be discussed when linked to dysfunction. For some participants, asking about sexual activity or sexual function appeared shaped by both age and gender:

I’d probably ask [about sexuality] more to women but then there probably is that cutoff with age and no arbitrary reason in my head, of what an age is […], over 60 maybe even. But then for men I probably would ask it up to any point, but the question would be more about achieving an erection rather than relating into sexual activity. *(George, 29, GP Registrar)*

This account suggests that women’s sexuality was more likely to be treated as age-limited, whereas men’s sexual health remained more clinically visible, although often through a narrower biomedical focus on erectile function.

Many participants noted that sexual health concerns typically arose in the context of menopause, prostate issues, or medication side effects. These clinical prompts created opportunities, however several participants noted that conversations were often medically focused and formed part of a brief checklist. Matthew described sexuality arising during menopause-related discussions: Definitely a lot with kind of menopause. It’s just one of the tick boxes, you know, when you’re asking females about that. So, it [sexuality] would come up, but it’s usually fairly brief conversation about [menopause]. *(Matthew, 43, GP)*

Participants often described these conversations as medicalised, framed around hormones or discomfort rather than intimacy. Similarly, men’s concerns were more easily legitimised when linked to medical comorbidities:

[…] people that got like cardiovascular risk factors like high blood pressure, raised cholesterol, etc, and they’re sort of males who sort of having problems like with intimacy then it’s almost easier when you can relate it back to an underlying physical cause that’s affecting other areas of their health. *(Ophelia, 28, GP)*

Some participants noted that although physical symptoms could lead to deeper emotional discussions, these were not pursued unless prompted by patient distress. Thus, while physical issues created clinically acceptable entry points, they did not guarantee a holistic approach to sexual wellbeing.

#### Theme 3: guidelines in the shadows: navigating the gaps between formal guidance and real-world practice

This theme, explored across two subthemes, reveals how participants relied on instinct and informal learning in the absence of clear guidance on sexuality in OAs.

**Learning in the trenches: how experience shapes conversations.** Most participants described developing their communication strategies through personal experience rather than formal training. The ‘learning by doing’ approach could be effective but inconsistent:

I think I’ve learned with time on my own, I have not had specific training for it […]. think doctors are not trained, staff are not trained as such, to talk about these things. *(Natasha, 45, GP Registrar)*

Similarly, Heidi described that ‘most’ learning came from practice rather than formal education:

We didn't get much training specifically on sexual health in older adults during medical school. Most of what I've learned comes from on-the-job experience. *(Heidi, 31, GP)*

For some, confidence in discussing sexual health grew with repeated exposure, as familiarity with these conversations helped to reduce discomfort. However, the absence of structured training meant that not all participants perceived they had equal opportunities to develop these skills and many consulted peers for advice:

I do speak to senior colleagues like other GPs or even friends [...] who are training to see how they approach [sexuality with OAs]. *(Prue, 32, GP Registrar)*

While shadowing senior colleagues could provide valuable insights, many participants felt that it was not always a reliable way to learn best practice. The findings suggest that sexual health discussions often depended on participants’ personal comfort and their consultation style, making observational learning highly variable. Some participants talked about how they lacked exposure to complex cases, leaving them feeling ill-prepared when faced with sensitive patient concerns. Without formal instruction, confidence in discussing sexual health remained inconsistent, contributing to variation in patient care.

**The gaps we feel and guidelines on autopilot.** Participants were asked about their awareness and use of guidance for discussing sexuality with OAs. All were unaware of formal guidance specific to this area. Where guidance was discussed more generally, it was often described as narrow, medically focused and disconnected from real-world clinical needs. This lack of formal direction contributed to widespread uncertainty, reliance on instinct and inconsistent practices, particularly in addressing broader psychological and relational dimensions of sexual wellbeing:

The very fact of having guidelines would be useful in the sense that it would be putting something out there. It’s almost, it’s so invisible, that’s why it doesn’t get discussed. *(Juliette, 62, MH Nurse)*

Most participants shared that under time pressure, formal guidance was rarely consulted during real-time consultations:

We have to look out realistically how many of us are going to look at a guideline or a resource before we initiate a conversation. *(Amelia, 38, Senior MH Practitioner)*

Current resources were also described as offering little support for managing clinician discomfort or patient resistance:

[…] how best to approach that or if they just, you know, outrightly say: ‘I’m not comfortable in discussing that with yourself.’ *(Prue, 32, GP)*

The absence of strategies for managing patient resistance was viewed as a significant shortcoming. Many participants stressed that emotionally charged or taboo topics required more than clinical knowledge and suggested guidance should account for interpersonal and cultural dynamics.

## Discussion

This study examined how primary care HCPs navigated discussions about sexuality with OAs. It explored when these conversations occurred or were avoided and the role (if any) clinical guidelines had in shaping these interactions. Although later-life sexual wellbeing is well established in literature [2, 4, 13, 19], participants described sexuality as rarely addressed in their own routine practice. Across accounts, emotional discomfort, gendered assumptions and perceived absence of structured support shaped how and when conversations occurred.

### Navigating psychological and systemic silence

Previous research has shown that clinicians often hesitate to address sexuality with OAs unless prompted by ‘natural openers’, such as physical symptoms or medication side effects [2, 14]. In this sample, reliance on biomedical cues appeared to be a strategic response to uncertainty and perceived risk. Earlier work has identified these cues as legitimising tools for difficult conversations [6, 14]. In their systematic review, Dyer and Das Nair [14] suggest that these strategies help preserve emotional distance and consultation flow. The present findings extend this by showing how the same cues may also reframe sexuality as a narrowly medical issue, rather than as part of lived experience in later life [14, 46].

Avoidance was often a professional response to structural constraints, echoing findings by Gott *et al.* [2, 13]. Participants in this study described feeling unprepared for unexpected disclosures, particularly in the absence of clear referral pathways [12]*.* This uncertainty was compounded by time pressures, limited training and absence of formal guidance, reinforcing a system in which emotional and relational concerns were deprioritised [5, 21, 26].

This dynamic reflected emotional labour [47], particularly the work involved in navigating OA sexuality as a topic vulnerable to taboo, embarrassment, misinterpretation and uncertainty about professional boundaries. Discomfort appeared to be shaped by the context, including patient age and gender, whether sexuality could be linked to a biomedical concern and whether participants felt they had sufficient training or guidance to support the conversation. Hinchliff *et al.* [19] suggest that such emotional competencies are often assumed rather than taught. Consistent with this, participants’ accounts indicated that the burden of managing discomfort and navigating sensitive topics often fell on individual clinicians, despite limited formal support. In this context, silence may have functioned as a form of institutional gatekeeping, shaping which topics become legitimised within care settings [6].

Several participants also described strategies that helped create more open conversations, including open-ended questions and normalising statements such as ‘I ask all my patients about this’. These approaches reflect the trust-building described by Gewirtz-Meydan *et al.* [48]. However, in this study, they were applied inconsistently and often depended on clinicians’ confidence, available time and rapport with the patient. Even when sexuality was discussed, conversations tended to remain focused on biomedical issues such as erectile dysfunction or menopause [6, 18], suggesting that medical cues not only initiated, but also constrained the scope of discussion [6, 46].

Participants further reported that OAs rarely initiated these conversations themselves, often waiting for professional cues to signal safety or legitimacy. This supports Gewirtz-Meydan *et al.*’s [48] observation that such discussions are shaped by perceived assumptions and expectations on both sides. In the present study, silence was not framed simply as a communication problem or a lack of interest. Rather, participants described a healthcare context that gave limited priority to emotional wellbeing and offered little structural support for engaging with sexuality beyond a biomedical lens [4, 14].

### Whose sexuality matters: gendered priorities

Gendered assumptions shaped how participants approached sexual health with OAs. Many described greater discomfort when speaking with patients of a different gender, often because of concerns about causing offence or being misunderstood. Fileborn *et al.* [25] highlight reluctance amongst female patients to raise such concerns with male clinicians, while Bauer *et al.* [4] note that many OAs expect professionals to initiate these conversations. Current findings suggest this hesitation was mutual, shaped not only by personal unease but also by uncertainty about how such conversations might be perceived [2, 12, 19]. These patterns reflect earlier work by Gott *et al.* [2] and Hinchliff *et al.* [19] and suggest that some clinicians experienced cross-gender discussions as interpersonally uncertain, with intentions and boundaries harder to interpret.

Participants often felt more at ease discussing sexual health with patients of the same gender. In contrast, conversations with patients of another gender were often avoided because of concern about misinterpretation. Several female participants described particular unease when older male patients raised issues related to sexual function. These conversations were sometimes interpreted as containing ‘hidden agendas’ or ambiguous undertones and raised concerns about professional boundaries [6, 46], suggesting that discomfort was not simply about sexual health itself, but about the interpersonal context in which it was raised. Clinicians had to manage not only the content of the interaction, but also their own emotional responses and concerns about how they might be perceived. While emotional labour has previously been discussed in the context of sexual health [47, 49], the present findings suggest that this work becomes especially demanding when clinicians feel exposed or uncertain, particularly in the absence of clear institutional support [22].

At the same time, participants were often more likely to raise sexuality with older male patients when framed biomedically, particularly through erectile dysfunction or cardiovascular risk, as reflected in Gewirtz-Meydan *et al.*’s findings [48]. In contrast, female sexuality, especially beyond menopause, was often seen as less relevant. One participant described helping an 80-year-old patient *‘change her perspective’* after she disclosed romantic feelings, shifting the interaction from acknowledgement to redirection. This aligns with previous research on marginalisation of older women’s sexual desire [18] and offers further insight into how such marginalisation may be enacted in everyday clinical encounters.

### Guidance in theory, gaps in practice

A recurring theme was the perceived absence of relevant and accessible guidance to support participants in addressing sexual health with OAs. None of the participants were aware of protocols specific to this group. Where guidance was recalled, it was typically perceived as general or oriented towards younger populations [14, 26]. Although models such as PLISSIT [32] offer structured approaches to initiating sexuality conversations, no participants referenced it, indicating gaps in dissemination, uptake and institutional integration [22, 26]. These accounts are consistent with observation that structured frameworks often fail to translate into routine practice due to systemic and institutional barriers [6]. The present findings further suggest that emotional readiness and contextual relevance are also central to whether such models are adopted meaningfully.

In the absence of structured guidance, participants relied on heuristics [23], peer advice or trial-and-error strategies, practices that contribute to variation in care [2, 6, 11]. Although such behaviours have previously been viewed as reactive [2], the present accounts suggest they are often shaped by emotional uncertainty and lack of institutional support, rather than by clinical improvisation alone.

Electronic prompts were mentioned frequently, particularly for topics such as menopause or erectile dysfunction, but were often described as being used mechanically or skipped altogether [11, 14]. This appeared to limit their usefulness in supporting meaningful conversations. Participants viewed these tools as overly biomedical and ill-equipped to address relational and emotional complexity of later-life sexuality, which in turn pushed practice back toward informal strategies. Reliance on symptom-based prompts further reinforced the idea that sexuality becomes clinically legitimate only when it presents as a medical problem.

Participants emphasised the need for guidance that moves beyond symptom checklists to address the psychosocial and cultural dimensions of sexuality, including communication strategies and emotionally attuned responses to complex disclosures [11]. This supports Davis and Weaver’s [26] findings that many interventions fail to equip professionals for the relational, emotional and communicative aspects of sexuality discussions. Current findings emphasise that even well-designed tools are ineffective without emotional literacy and experiential learning. Participants noted that the emotional weight of these disclosures cannot be managed through checklists or flowcharts alone [46].

The concept of self-efficacy [20] offers a useful lens for understanding these gaps. Knowledge alone may be insufficient if clinicians do not feel able to apply it in practice [20, 26]. Without emotionally attuned training and sustained system-level support, frameworks like PLISSIT are unlikely to move beyond theory into practice [22]. These accounts illustrated how the absence of such support can leave later-life sexual health marginalised, particularly in systems that prioritise clinical efficiency over relational care.

### Implications for practice

Participants’ accounts suggest that training programmes should better reflect the complexity of these conversations by including interpersonal communication, boundary management, cultural competence and reflection on gendered ageism [22, 26]. Such training may help HCPs recognise when assumptions about ageing, gender and menopause shape clinical conversations, preventing older women’s concerns from being dismissed as a natural part of ageing while broadening discussions with older men beyond erectile function. Opportunities for practice, such as brief modules, case-based learning, workshops or roleplays, could be integrated into continuing professional development to help HCPs build confidence and consistency [19, 26].

Training alone is unlikely to be sufficient without structural support. Existing frameworks, including PLISSIT [32], need to be embedded into routine practice rather than left as optional tools [22, 25]. Professional guidance should move away from framing later-life sexuality as an isolated issue and instead include it within standard care pathways aligned with the needs of OAs [22, 26].

Making sexual health a routine part of care could reduce missed opportunities and help OAs feel more comfortable bringing up their concerns [25, 26]. Integrating open-ended questions about intimacy or relationships into routine health assessments [14, 25, 26] could help break mutual silence around sexuality without substantially extending consultations [6, 14, 19]. For example, embedding brief scripts or reflective prompts into digital patient records may support clinicians in normalising discussions of sexuality, even under time constraints. Any resources developed for HCPs should therefore be brief, easy to use within time-limited consultations and include guidance on responding to patient discomfort or reluctance.

### Strengths and limitations

A key strength of this study is its focus on real-world clinical experiences, with qualitative data capturing how personal discomfort, time constraints and structural limitations shaped reported participant behaviour. Findings may have practical relevance for training, service delivery and policy.

Limitations include reliance on self-reports; participants may have presented themselves as more confident or proactive than they are in practice [50]. The sample may not represent all settings or professional roles. Despite recruitment efforts, physician associates, practice nurses, clinical pharmacists, allied health professionals and psychological practitioners were not represented. Service users were not directly involved, so finding reflects professionals’ rather than OAs’ perspectives. Demographic homogeneity (notably gender and age) and limited exploration of sexual orientation and gender identity may have limited understanding of intersecting assumptions about age and sexuality. Findings should therefore be interpreted as contextual to this sample and may not generalise to other settings.

### Future research implications

Future work could include OAs’ perspectives on sexuality discussions in primary care, particularly in the context of remote consultations and evolving care models [18]. Examining sexuality within chronic condition management, such as diabetes, cardiovascular disease or neurological injury, could explore whether integrating sexual wellbeing into routine management normalises the topic and improves care. Finally, evaluating practical interventions such as digital prompts or visual aids and examining approaches in secondary and specialist care, may inform more confident primary care practice.

## Conclusion

Although sexuality remains important in later life, it remains inconsistently addressed in primary care. HCPs experience several barriers, including discomfort, time pressure and a lack of clear guidance. There are opportunities to improve practice through targeted training, integrating sexual health into routine assessments and revising official guidelines to include OAs. By addressing these gaps, HCPs can take a more active role in supporting sexual wellbeing in later life.

## Footnotes

1
*Note:* Participants’ names have been anonymised using alphabetical pseudonyms. Each participant was assigned a name beginning with a successive letter of the alphabet (e.g. Participant 1 was given a random name starting with letter ‘A’).
